# Association between domesticated animal ownership and *Plasmodium falciparum* parasite prevalence in the Democratic Republic of the Congo: a national cross-sectional study

**DOI:** 10.1016/S2666-5247(23)00109-X

**Published:** 2023-07

**Authors:** Camille E Morgan, Hillary M Topazian, Katerina Brandt, Cedar Mitchell, Melchior Mwandagalirwa Kashamuka, Jérémie Muwonga, Eric Sompwe, Jonathan J Juliano, Thierry Bobanga, Antoinette Tshefu, Michael Emch, Jonathan B Parr

**Affiliations:** a Department of Epidemiology, Gillings School of Global Public Health, University of North Carolina at Chapel Hill, Chapel Hill, NC, USA; b Department of Geography, University of North Carolina at Chapel Hill, Chapel Hill, NC, USA; c Institute for Global Health and Infectious Diseases, University of North Carolina at Chapel Hill, Chapel Hill, NC, USA; d Division of Infectious Diseases, Institute for Global Health and Infectious Diseases, University of North Carolina at Chapel Hill, Chapel Hill, NC, USA; e MRC Centre for Global Infectious Disease Analysis, Imperial College London, London, UK; f School Of Public Health, University of Kinshasa, Kinshasa, DR Congo; g Department of Tropical Medicine, Faculty of Medicine, University of Kinshasa, Kinshasa, DR Congo; h Programme National de La Lutte Contre Le SIDA, Kinshasa, DR Congo; i Programme National de La Lutte Contre Le Paludisme, Kinshasa, DR Congo; j Faculty of Medicine, School of Public Health, University of Lubumbashi, Kinshasa, DR Congo

## Abstract

**Background:**

Domesticated animal ownership is an understudied aspect of the human environment that influences mosquito biting behaviour and malaria transmission, and is a key part of national economies and livelihoods in malaria-endemic regions. In this study, we aimed to understand differences in *Plasmodium falciparum* prevalence by ownership status of common domesticated animals in DR Congo, where 12% of the world's malaria cases occur and anthropophilic *Anopheles gambiae* vectors predominate.

**Methods:**

In this cross-sectional study, we used survey data from individuals aged 15–59 years in the most recent (2013–14) DR Congo Demographic and Health Survey and previously performed *Plasmodium* quantitative real-time PCR (qPCR) to estimate *P falciparum* prevalence differences by household ownership of cattle; chickens; donkeys, horses, or mules; ducks; goats; sheep; and pigs. We used directed acyclic graphs to consider confounding by age, gender, wealth, modern housing, treated bednet use, agricultural land ownership, province, and rural location.

**Findings:**

Of 17 701 participants who had qPCR results and covariate data, 8917 (50·4%) of whom owned a domesticated animal, we observed large differences in malaria prevalence across types of animals owned in both crude and adjusted models. Household chicken ownership was associated with 3·9 (95% CI 0·6 to 7·1) more *P falciparum* infections per 100 people, whereas cattle ownership was associated with 9·6 (–15·8 to –3·5) fewer *P falciparum* infections per 100 people, even after accounting for bednet use, wealth, and housing structure.

**Interpretation:**

Our finding of a protective association conferred by cattle ownership suggests that zooprophylaxis interventions might have a role in DR Congo, possibly by drawing *An gambiae* feeding away from humans. Studies of animal husbandry practices and associated mosquito behaviours could reveal opportunities for new malaria interventions.

**Funding:**

The National Institutes of Health and the Bill & Melinda Gates Foundation.

**Translations:**

For the French and Lingala translations of the abstract see Supplementary Materials section.

## Introduction

Despite substantial research investment and attention, malaria cases and deaths are increasing in high-burden countries, with an estimated 247 million cases and 619 000 deaths worldwide in 2021.^1^ 96% of these deaths occurred in the WHO African region, where the most virulent species of the parasite that causes malaria, *Plasmodium falciparum*, predominates.^1^ Malaria infections involve a complex interplay of environment, pathogen, vector and human host biology, and human behaviour. Substantial research and public health programming have been allocated to examining parasite–vector–host biology, but many questions remain about how local environmental factors influence *P falciparum* transmission.

DR Congo accounts for the second highest percentage of malaria cases worldwide (12%) and 54% of the cases in central Africa.^2^ Anthropophilic *Anopheles gambiae* predominate, with *An gambiae* sensu stricto most commonly found, followed by *Anopheles coluzzii*.^3^ As the second largest country in Africa by area and one of the most biodiverse regions of the world, DR Congo has high ecological and population diversity across a large geographical area.^4^ Although previous research identified demographic and environmental factors associated with low *P falciparum* prevalence, such as wealth, modern housing, and low agricultural cover,^5^, ^6^ the relationship between livestock and farm animal ownership—a key source of income and the country's overall economy^7^—and *P falciparum* infection remains undefined.

Human–animal interactions influence local disease ecology, and the effect of livestock on malaria is an important question given that the introduction of livestock as zooprohylatic decoys (ie, approaches to draw mosquito bites away from humans and towards animals) or as a means of using veterinary endectocides has been proposed as an intervention (ie, a One Health strategy) to reduce malaria infections.^8^, ^9^ A 2015 systematic review found that zooprophylaxis shows promise in reducing malaria infections, particularly in areas where zoophilic mosquito vectors predominate, livestock are kept away from areas where humans sleep, and bednets are used.^10^ Other studies have found that proximity of cattle to human dwellings is protective, such as in a region of Tanzania where *Anopheles arabiensis* dominates,^11^ whereas other studies have found that cattle have no effect on malaria transmission, such as in The Gambia where *An gambiae* sensu stricto*, An melas*, and *An arabiensis* predominate.^12^ In other areas, livestock have been found to increase malaria prevalence (ie, zoopotentiation), potentially by creating an environment near the household that is hospitable to mosquito breeding.^13^ Thus, the effect of livestock ownership on malaria risk is unclear but appears to be context specific.

**Research in context**

**Evidence before this study**

We searched PubMed using search terms (“domesticated animal” OR “household animal ownership” OR “animal”) AND (“malaria” OR “plasmodium”) for any articles in any language published from database inception to May 24, 2022. None of the studies identified investigated this question in DR Congo. Several studies have evaluated aspects of zooprophylaxis and zoopotentiation for malaria control in Ethiopia, The Gambia, Ghana, Mozambique, Senegal, Tanzania, and Zambia, or included specific animals owned in malaria risk factor analyses (eg, in Burkina Faso, Ethiopia, Guinea-Bissau). The evidence is mixed across these settings: in Macha, Zambia and southern Tanzania, where the moderately zoophilic *Anopheles arabiensis* predominates, cattle ownership was protective against malaria infection, but had no effect in anthropophilic *Anopheles gambiae* sensu stricto regions of The Gambia or zoophilic *An arabiensis* regions of Ethiopia. Chicken ownership appears protective in Ethiopia, but chicken DNA is not frequently found in bloodmeals across contexts. In a feeding analysis of 1886 *An gambiae* sensu lato in Senegal, 37·1% of *An gambiae* sensu lato feedings involved single-animal blood meals and a similar proportion were observed to be single human origin. A 2015 systematic review of zooprophylaxis for malaria control concluded that zooprophylaxis has potential as an effective strategy—particularly in contexts of zoophilic vectors, large distances between where animals are kept at night and where humans sleep, and frequent bednet use—but more evidence is needed for specific environmental conditions. Further, increasing use of insecticide-treated bednets is predicted to increase feeding on cattle by anthropophilic *An gambiae* sensu stricto vectors.

**Added value of this study**

Our study investigates whether zooprophylaxis could offer protection against malaria in DR Congo, where *Plasmodium falciparum* remains highly prevalent despite ongoing control efforts and where the anthropophilic *An gambiae* sensu stricto vector is widespread and the anthropophilic *Anopheles coluzzi* is also common. In the largest analysis of malaria zooprophylaxis in Africa conducted to date, we found a protective effect of cattle ownership against *P falciparum* infection, even after accounting for household wealth, treated bednet use, agricultural land, and modern housing. This finding raises the possibility that cattle kept near household sleeping quarters could draw mosquitos away from humans and reduce household *P falciparum* transmission risk. We also found increased *P falciparum* infection prevalence with chicken ownership. Although rare in this population, ownership of horses, donkeys, or mules was also associated with an increased *P falciparum* prevalence; however, this result was not statistically significant in adjusted models. Goat, sheep, pig, and duck ownership did not result in a difference in infection prevalence.

**Implications of all the available evidence**

These findings suggest that cattle ownership in the household compound offers protection against *P falciparum* infection and that chicken ownership confers risk of *P falciparum* infection in DR Congo. There might be a role for zooprophylaxis in DR Congo and other settings where *P falciparum* transmission via *An gambiae* vectors is high. Future studies of animal ownership and husbandry practices could reveal opportunities for novel malaria control interventions, such as optimising distance between humans and household animals combined with the use of veterinary endectocides.

Feeding preferences of the mosquito species are an important determinant of an animal's zooprophylaxis or zoopotentiation effect on human malaria infection. These effects are evaluated through analysis of mosquito blood meal content to establish the proportion from humans (ie, human blood indices). In Tanzania, human blood meals have been found to be less frequent in *An arabiensis* and *Anopheles funestus* sensu lato vectors where livestock are present in the household compared with households without livestock, whereas human blood meals in *An gambiae* sensu stricto appeared unaffected by the presence of household livestock.^14^ Although this finding is consistent with previous research establishing anthropophilic preferences of *An gambiae* sensu stricto and zoophilic tendencies of *An arabiensis* and *An funestus*,^15^ human blood indices are highly variable. The human blood indices of *An arabiensis* ranges from 0% to 80% across surveys, suggesting adaptability of the zoophilic vector to feed across hosts, including an increased proportion from humans.^16^ Vector biting preferences are further predicted to change with malaria control measures. Anthropophilic *An gambiae* sensu stricto might be driven towards a preference for cattle feeding with increasing use of insecticide-treated bednets.^17^

This adaptability of mosquito vectors underscores the need to move beyond human-centred malaria prevention, and consider the broader environment, including non-human hosts. Improved understanding of the relationship between household animals and malaria prevalence in DR Congo is needed to identify One Health strategies that might reduce DR Congo's high malaria burden. In this study, we aimed to investigate the association between *P falciparum* prevalence and ownership status of common domesticated animals in DR Congo.

## Methods

### Study design and population

In this population-based, nationally representative, cross-sectional study with a two-stage cluster design, we investigated the association between animal ownership and *P falciparum* infection using the most recent (2013–14) DR Congo Demographic and Health Survey (DHS) and previously collected dried blood spots.^18^

In the first stage, 540 sampling clusters across 26 DR Congo provinces were sampled, and in the second stage, households were randomly sampled within each cluster. Men (aged 15–59 years) and women (aged 15–49 years) from the selected households were offered enrolment, including collection of a dried blood spot specimen. Consent for participation, specimen collection, and specimen analysis for other biomarkers was more than 95%.

Additional ethics approval was not required for this analysis of publicly available, de-identified data. The parent studies from which data used in this analysis were generated were approved by Kinshasa School of Public Health (ESP/CE/015/14) and the University of North Carolina at Chapel Hill Institutional Review Board (number 14-0077).

### Exposure, outcome, and covariate classification

We defined the exposure as a binary variable of each type of livestock, herd, or farm animal owned versus not owned by the household, including cattle; chickens; ducks; goats; horses, donkeys, or mules; pigs; and sheep. We defined the outcome as a binary variable reflecting quantitative real-time PCR (qPCR)-confirmed *P falciparum* infection identified using participant dried blood spot samples. As described elsewhere,^5^ DNA was extracted using Chelex 100 (Bio-Rad, Hercules, CA, USA) and evaluated using a qPCR assay targeting *P falciparum* lactate dehydrogenase with a limit of detection of 5–10 parasites per μL.^19^, ^20^ Gender was determined by self-report with binary male or female options. Bednet use was collapsed into a single variable comparing if the participant had slept under a long-lasting insecticidal treated bednet the previous night versus using an untreated bednet or no bednet. Wealth was operationalised as quintiles calculated by the DHS programme; briefly, these quintiles originate from a principal components analysis of household attributes reflecting the standard of living.^21^ Rural location was a binary categorisation determined by country administrators. Modern housing was dichotomised as a composite binary variable reflecting houses with floor, wall, and roof material that is more insulated and less penetrable by mosquitoes for inhabitants compared with traditional housing materials, as described elsewhere.^5^

### Statistical analysis

We used a directed acyclic graph (appendix 3 pp 2–5) to identify confounders and modifiers of the relationship between animal ownership and malaria. We then sought to reduce the model by comparing the change in estimate and precision across multiple iterations (appendix 3 pp 2–5). Confounders included in the adjusted models were gender, treated bednet use, wealth, rural location, and modern housing.

The survey did not include information on where animals were kept overnight, but we used past qualitative research to inform a subanalysis with cattle ownership—ie, households with fewer cattle might be more likely to keep animals within the household compound, as they are less likely to be stolen when kept near the household.^22^ As a small but unknown number of cattle can be feasibly kept on the compound, we tested the relationship between different thresholds of herd size and *P falciparum* prevalence. We also conducted a post-hoc analysis of different flock sizes for chickens.

We used linear binomial regression models with generalised estimating equations to account for the clustered data structure to estimate crude and adjusted prevalence differences in *P falciparum* infections between participants from households that owned each animal compared with participants from households that did not own each respective animal. We applied survey sample weights to provide estimates of the target population based on the sampling design. For subanalyses, we used the log and logit links to calculate prevalence ratios and odds ratios to enhance model convergence given small sample sizes. We calculated profile likelihood 95% CIs.

We conducted all analyses in R (version 4.0.3).

### Role of the funding source

The funders of the study had no role in study design, data collection, data analysis, data interpretation, or writing of the report.

## Results

A total of 17 703 participants were eligible for inclusion and 17 701 (99·9%) had non-missing values for all covariates (appendix 3 p 6), representing 18 091 individuals when weighted to the total DR Congo population. When weighted, 31·1% (95% CI 29·0–33·0) of participants included in the analysis had PCR-confirmed *P falciparum* malaria (table 1).

**Table 1:** Characteristics of the study population, accounting for survey sample weights

				Plasmodium falciparum infection*	No *P falciparum* infection*	**Overall**
Total				5625 (31·1%)	12 466 (68·9%)	18 091
Household members				6·70 (0·09)	6·77 (0·09)	6·75 (0·08)
Children aged <5 years in household				1·46 (0·03)	1·47 (0·03)	1·47 (0·02)
Age, years				28·2 (0·20)	30·4 (0·17)	29·7 (0·12)
Gender						
	Male			2901 (51·6%)	5685 (45·6%)	8586 (47·5%)
	Female			2724 (48·4%)	6781 (54·4%)	9505 (52·5%)
Slept under treated bed net previous night						
	Yes			2798 (49·7%)	6860 (55·0%)	9658 (53·4%)
	No			2827 (50·3%)	5606 (45·0%)	8433 (46·6%)
Modern housing						
	Yes			618 (11·0%)	2600 (20·9%)	3218 (17·8%)
	No			5007 (89·0%)	9866 (79·1%)	14 873 (82·2%)
Wealth quintile						
	Lowest			1222 (21·7%)	2111 (16·9%)	3333 (18·4%)
	Lower-middle			1198 (21·3%)	2285 (18·3%)	3483 (19·3%)
	Middle			1322 (23·5%)	2352 (18·9%)	3674 (20·3%)
	Upper-middle			1157 (20·6%)	2439 (19·6%)	3596 (19·9%)
	Highest			726 (12·9%)	3279 (26·3%)	4005 (22·1%)
Rurality						
	Rural location			3917 (69·6%)	7605 (61·0%)	11 522 (63·7%)
	Urban location			1708 (30·4%)	4861 (39·0%)	6569 (36·3%)
Agricultural land ownership						
	Yes			3744 (66·6%)	7169 (57·5%)	10 914 (60·3%)
	No			1879 (33·4%)	5297 (42·5%)	7176 (39·7%)
Livestock, herd, or farm animal ownership						
	Owns any animal			2993 (53·2%)	5923 (47·5%)	8917 (49·3%)
		Cattle		62 (1·1%)	300 (2·4%)	362 (2·0%)
			Median owned	4 (2–7)	3 (2–5)	3 (2–6)
		Chickens		2596 (46·2%)	4936 (39·6%)	7532 (41·6%)
			Median owned	5 (2–9)	4 (2–8)	5 (2–8)
		Horses†		10 (0·2%)	20 (0·2%)	31 (0·2%)
			Median owned	10 (3–10)	10 (3–10)	10 (3–10)
		Goats		1127 (20·0%)	2445 (19·6%)	3572 (19·8%)
			Median owned	2 (1–4)	2 (1–4)	2 (1–4)
		Sheep		209 (3·7%)	482 (3·9%)	690 (3·8%)
			Median owned	2 (1–4)	2 (1–3)	2 (1–3)
		Pigs		481 (8·6%)	1134 (9·1%)	1617 (8·9%)
			Median owned	2 (1–3)	2 (1–4)	2 (1–4)
		Ducks		597 (10·6%)	1129 (9·1%)	1726 (9·5%)
			Median owned	3 (2–5)	3 (2–5)	3 (2–5)

Data are mean (SD), median (IQR), or n (%).

* Infection confirmed by *P falciparum* lactate dehydrogenase quantitative PCR.

† Donkeys, horses, or mules.

This subset was similar to the overall DHS cohort that has been described elsewhere,^5^ but, in brief, participants in this analysis with *P falciparum* infection tended to be slightly younger than those who were *P falciparum* negative, and more participants with *P falciparum* infection were men than women (table 1). About half of participants had slept under a treated bednet the previous night, with a lower percentage of participants with *P falciparum* infection having slept under a treated bednet than participants without *P falciparum* infection (table 1). Only about a tenth of those with *P falciparum* infection had modern housing, compared with two-fifths of those without *P falciparum* infection (table 1). Of *P falciparum*-positive participants, the smallest proportion were in the highest wealth quintile (12·9%), whereas of *P falciparum*-negative participants, the largest proportion were in the highest wealth quintile (26·3%; table 1). Living in a rural area was more common among *P falciparum*-positive participants than *P falciparum*-negative participants, as was agricultural land ownership (table 1).

**Figure 1:**
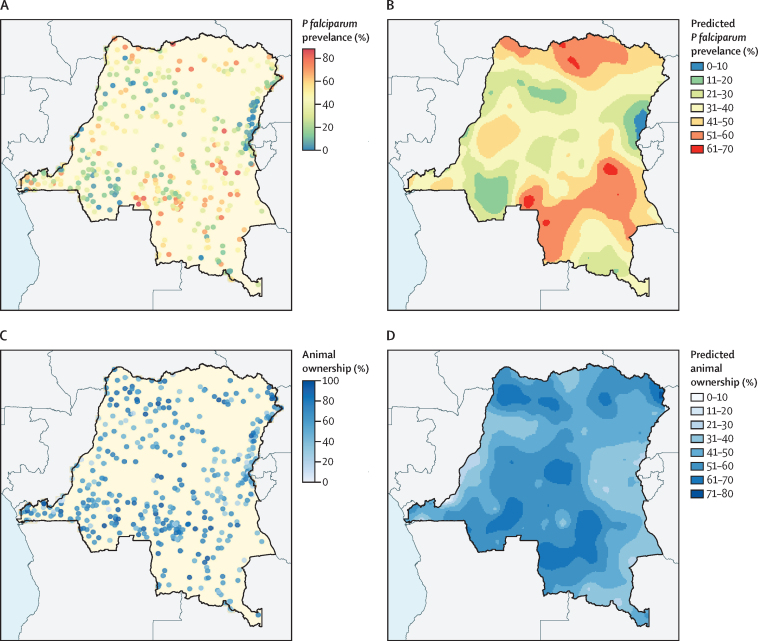
Geographical prevalence of animal ownership and *Plasmodium falciparum* infection in DR Congo Geographical prevalence of *P falciparum* and animal ownership by Demographic and Health Survey cluster (A, C). Predicted regional distribution of *P falciparum* and animal ownership (B, D). Respondents with missing data on animal ownership or geographical location were excluded.

About half of all participants lived in households that owned any domesticated animal (table 1). Chickens were the most common animal owned (41·6% of participants, 95% CI 38·9–44·0), followed by goats (19·8%, 17·9–22·0). Only 2·0% (1·4–3·0) of all participants lived in households that owned cattle, and 0·2% (0·1–0·2) lived in households that owned horses, donkeys, or mules. Except for horses, donkeys, or mules, ownership of each animal type followed a right-skewed distribution, with most individuals living in households with five or fewer of a given animal (appendix 3 p 7). The overlap of animals owned is depicted in appendix 3 (p 8). *P falciparum* prevalence and animal ownership varied spatially, with higher proportions of animal ownership seen in the rural south-central and northern regions than in other regions of the country (figure 1; appendix 3 pp 9–10).

**Figure 2:**
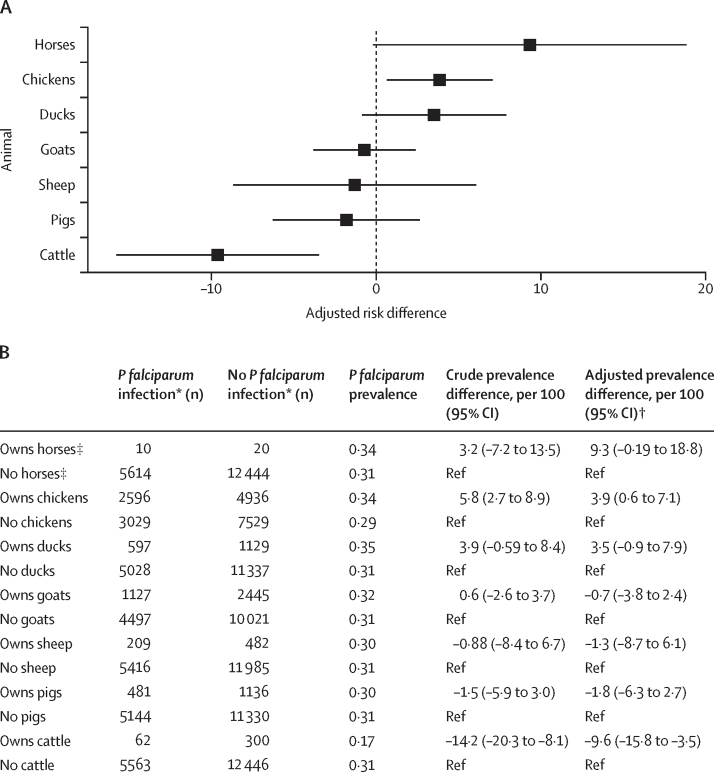
Crude and adjusted associations between animal ownership and *Plasmodium falciparum* infection (A) Adjusted *P falciparum* prevalence difference (95% CI) by each animal owned versus not owned. (B) Weighted counts and crude and adjusted associations for each animal and *P falciparum* infection. Prevalence difference associations were estimated with generalised estimating equation models to account for clustering within households. Survey weights are incorporated. *Infection confirmed by *P falciparum* lactate dehydrogenase quantitative PCR. †Adjustment set includes gender, treated bednet, modern housing, wealth, and rurality. ‡Donkeys, horses, or mules.

Participants from households that owned chickens had 3·9 (95% CI 0·6 to 7·1) more *P falciparum* infections per 100 people compared with participants from households without chickens, after adjusting for gender, treated bednet use, modern housing, wealth, and rurality (figure 2). Participants from households that owned cattle had 9·6 (95% CI –15·8 to –3·5) fewer *P falciparum* infections per 100 people compared with participants from households without cattle, after adjusting for the same variable set. The remaining animal types (ie, ducks, goats, sheep, pigs, and horses, donkeys, or mules) did not show a significant protective or harmful association with *P falciparum* infection (figure 2).

When both chickens and cattle were owned by a household, the protective association of cattle ownership remained (appendix 3 p 11). *P falciparum* prevalence among individuals living in households with chickens and cattle was 20% (appendix 3 p 11), similar to the prevalence among all cattle owners (17%), but lower than the prevalence among all chicken owners (34%; figure 2).

In crude models, participants in households with between one and four cattle had 17·8 fewer (95% CI –24·8 to –10·8) *P falciparum* infections per 100 people compared with households that owned no cattle. We observed no signficant association between ownership of five or more cattle and *P falciparum* infection (table 2). When the threshold of ten cattle was used, a similar prevalence difference was observed for owning one to nine cattle and ten or more cattle (data not shown). These associations were similar in adjusted models, suggesting a beneficial effect of owning fewer than five cattle on the risk of *P falciparum* infection and no evidence of an effect when five or more cattle are owned (data not shown). All chicken flock sizes (ie, 1–4, 5–9, 10–14, and ≥15 chickens) were associated with increased *P falciparum* prevalence compared with households that owned no chickens in unadjusted models, with an increasing prevalence ratio with more chickens owned (table 2). All except the smaller two flock sizes (<10 chickens) were significantly associated with increased *P falciparum* prevalence in adjusted models (table 2).

**Table 2:** Crude and adjusted associations between number of cattle owned and *Plasmodium falciparum* infection, and between number of chickens owned and *P falciparum* infection

	**n**	**Crude prevalence difference, per 100 (95% CI)**	**Crude prevalence ratio (95% CI)**	Adjusted prevalence ratio (95% CI)*
**Cattle**				
≥5 cows or bulls	124	−7·3 (−16·0 to 1·4)	0·77 (0·54 to 1·10)	0·98 (0·66 to 1·46)
1–4 cows or bulls	238	−17·8 (−24·8 to −10·8)	0·43 (0·26 to 0·72)	0·44 (0·26 to 0·75)
No cows or bulls	17 726	Ref	Ref	Ref
**Chickens**				
≥15 chickens	753	11·5 (5·1 to 18·0)	1·40 (1·18 to 1·66)	1·40 (1·18 to 1·66)
10–14 chickens	792	8·8 (4·0 to 13·6)	1·31 (1·14 to 1·50)	1·27 (1·10 to 1·46)
5–9 chickens	2258	5·0 (0·9 to 9·1)	1·17 (1·03 to 1·34)	1·11 (0·98 to 1·26)
1–4 chickens	3730	4·4 (0·8 to 8·1)	1·15 (1·03 to 1·30)	1·06 (0·94 to 1·19)
No chickens	10 557	Ref	Ref	Ref

Prevalence associations were estimated with generalised estimating equation models to account for clustering within households, with survey weights applied.

* Adjustment set includes sex, treated bednet, modern housing, wealth, and rurality.

The protective association between cattle ownership and *P falciparum* infection remained significant after assessing agricultural land ownership as a modifier (appendix 3 p 12). A protective association was observed among participants with land, while a non-significant association was found among participants without land usable for agriculture (appendix 3 p 12).

## Discussion

To our knowledge, this study is the largest investigation of household animal ownership and *P falciparum* prevalence done in Africa to date. We found that cattle ownership confers a protective association with *P falciparum* infection in DR Congo, resulting in 9·6 fewer *P falciparum* infections per 100 people. This protective association held after accounting for wealth, housing structure, bednet use, and modification by agricultural land. We found ownership of chickens to be associated with an increased prevalence of *P falciparum* infection. We did not observe a significant relationship between *P falciparum* prevalence and horse, donkey, or mule; goat; sheep; duck; and pig ownership. In a setting where cattle ownership and chicken ownership are common across the country, our findings suggest new opportunities for integrated vector management in DR Congo.

The protective association of cattle ownership was strongest when the herd size was small. Although the DHS did not collect information about where animals were kept overnight, we used herd size as a proxy for where cattle might be kept overnight, based on past research in DR Congo indicating that households with a small number of cattle were more likely to keep animals within the household compound.^22^ Keeping cattle closer to the household's sleeping area could yield an increased zooprophylactic effect by drawing mosquito feeding away from humans. This effect requires further investigation given the modification observed by agricultural land ownership. Previous research in *An arabiensis* or *Anopheles pharoensis* regions of Ethiopia found that large cattle herds (>20 cows per human) did not provide a zooprophylactic effect;^23^ however, other research has shown that increased cattle population density appeared to reduce vectors entering indoor areas of households, thus being potentially protective against malaria.^24^ In other settings, household cattle ownership appeared protective, and was associated with reduced odds of malaria infection in regions of Zambia with *An Arabiensis*.^25^ In Ghana, cattle being within 20 meters of humans was associated with a 66% reduction in the number of *An gambiae* sensu stricto landings on humans.^26^ Together, these findings highlight the context-dependent interplay among mosquito vectors, environment, and animal husbandry, and provide a biological basis for the protective association of cattle observed in this study.

We further observed that household chicken ownership was associated with increased *P falciparum* prevalence, and that prevalence increased with flock size. *An gambiae* sensu lato bloodmeals from chickens are found least frequently,^27^ suggesting that chickens have less zoophilic potential than do other household animals. Our finding contrasts with other studies that found a protective effect of chicken ownership in the context of *An arabiensis* vectors in Ethiopia.^28^ Although chickens were not a desired source of bloodmeals for female *An arabiensis*, volatile compounds secreted by chickens acted as medium-to-long range deterrents for female *An arabiensis*.^28^ Our contrasting findings have a few potential biological explanations. One is that the olfactory and other volatile compounds secreted by chickens might have little effect on *An gambiae* sensu stricto species, a common vector in DR Congo. Second, living conditions in DR Congo for chickens might provide additional mosquito breeding habitats, amplifying vector abundance. This amplification could be particularly plausible given the increasing prevalence ratios with larger flock sizes.

Although we did not observed a significant increase in *P falciparum* prevalence in households with horses, donkeys, or mules compared with households without these animals, ownership of these animals was rare and this estimate was imprecise. In contexts with *An gambiae*, donkeys have been associated with reduced malaria in univariate analyses,^29^ but because these animals are not frequently owned in DR Congo, interventions focused on them might not be widely effective. We estimated no noticeable association between *P falciparum* infection with each of the remaining animals (goats, sheep, ducks, and pigs). In contexts with anthropophilic *An gambiae* sensu stricto and *An funestus*, pig ownership has been found to be associated with increased odds of positivity on malaria rapid diagnostic test among children (aged 1–15 years).^30^ In *An arabiensis* regions of Ethiopia, sheep and goats have not been associated with an increased risk of *P falciparum* infection.^31^

This study has a few notable limitations. First, the cross-sectional design limits investigation of temporal trends between the exposure and outcome, malaria seasonality, and migratory patterns of some animals, particularly cattle. Second, although multiple iterations of the models resulted in estimates of similar magnitude and direction as the models presented, unmeasured confounding or measurement error of the self-reported exposure could nonetheless induce bias. Third, refusal to participate or provide a blood sample could result in selection bias; however, refusal rates were very low (<5%) across this nationally representative study and it is unlikely to substantially bias results. Finally, absence of information about household-specific animal husbandry restricts our ability to identify the behaviours or practices most amenable to intervention.

These findings suggest that interventions focused on household animals, particularly cattle and chickens kept in designated pens on the household compound, could have a role in DR Congo as a complement to current malaria control interventions. Future studies are needed to evaluate where animals are kept in relation to human sleeping quarters, as well as environmental and behavioural conditions in DR Congo that might affect the interplay between the vector, livestock, and human host. Promotion of husbandry practices found to be protective against malaria could complement ongoing malaria control activities in high-burden countries with dominant *An gambiae* sensu stricto vectors such as DR Congo.

## Data sharing

Data are available in a public, open access repository and upon request from the DHS programme (https://dhsprogram.com/methodology/survey/survey-display-421.cfm), including malaria PCR results. R code used for the analysis is available at https://github.com/IDEELResearch/animalaria.

## Declaration of interests

JBP reports research support from Gilead Sciences, non-financial support from Abbott Laboratories, consulting for Zymeron Corporation, and honoraria from Virology Education, all outside the scope of this work. JBP also reports malaria research support from WHO, unrelated to this work. All other authors declare no competing interests.

## Supplementary Materials

French translation of the abstract

Lingala translation of the abstract
