# Supplementary material for: Association between domesticated animal ownership and Plasmodium falciparum parasite prevalence in the Democratic Republic of the Congo: a national cross-sectional study
Source: Lancet Microbe. 2023 Jul;4(7):e516–23. doi: 10.1016/S2666-5247(23)00109-X (PMC10319634; doi:10.1016/S2666-5247(23)00109-X)
Supplement: French translation of the abstract [file mmc1.pdf]

THE LANCET
Microbe

Supplementary appendix 1

This translation in French was submitted by the authors and we reproduce it as supplied. It has not been peer reviewed. *The Lancet*'s editorial processes have only been applied to the original in English, which should serve as reference for this manuscript.

Cette traduction en français a été proposée par les auteurs et nous l'avons reproduite telle quelle. Elle n'a pas été examinée par des pairs. Les processus éditoriaux du *Lancet* n'ont été appliqués qu'à l'original en anglais et c'est cette version qui doit servir de référence pour ce manuscrit.

Supplement to: Morgan CE, Topazian HM, Brandt K, et al. Association between domesticated animal ownership and *Plasmodium falciparum* parasite prevalence in the Democratic Republic of the Congo: a national cross-sectional study. *Lancet Microbe* 2023; published online May 31. https://doi.org/10.1016/S2666-5247(23)00109-X.

Titre : Association entre la possession d'animaux domestiques et la prévalence du parasite Plasmodium falciparum en République démocratique du Congo : Une enquête nationale transversale

Camille E. Morgan, BSPH[1*], Hillary M. Topazian, PhD[2], Katerina Brandt, BA[3], Cedar Mitchell, PhD[4], Melchior Kashamuka Mwandagalirwa, MSPH[5], Jérémie Muwonga, PhD[6], Eric Sompwe, MD, PhD[7,8], Jonathan J. Juliano, MD[1,9], Thierry Bobanga, MD, PhD[10], Antoinette Tshefu, MD, PhD[5], Michael Emch, PhD[3], Jonathan B. Parr, MD, MPH[9]

<u>Résumé</u>

Contexte : La possession d'animaux de ferme domestiques par les ménages est un aspect peu étudié de l'environnement humain qui influence le comportement de piqûre des moustiques et la transmission du paludisme, et constitue un élément clé des économies nationales et des moyens de subsistance dans les régions où le paludisme est endémique. Centrée sur la République démocratique du Congo (RDC), où 12 % des cas de paludisme sont recensés dans le monde et où les vecteurs anthropophiles *Anopheles gambiae* prédominent, cette étude a cherché à comprendre les différences de prévalence de *Plasmodium falciparum* en fonction du statut de propriété de sept animaux domestiques courants.

Méthodes : En utilisant les données d'enquête des personnes âgées de 15 à 59 ans dans l'Enquête Démographique et de Santé (EDS) la plus récente (2013-14) de la RDC et la PCR quantitative en temps réel (qPCR) de Plasmodium effectuée précédemment, nous avons estimé les différences de prévalence de *P. falciparum* en fonction de la propriété des ménages de bovins, poulets, ânes / chevaux, canards, chèvres, moutons et porcs. Nous avons utilisé des graphes acycliques dirigés pour prendre en compte les facteurs de confusion tels que l'âge, le sexe, la richesse, les logements modernes, l'utilisation de moustiquaires imprégnées d'insecticide, la propriété de terres agricoles, la province et la situation rurale.

Résultats : Parmi les 17 701 participants ayant des résultats de qPCR et des données de covariables, près de la moitié d'entre eux possédaient un animal domestique, nous avons observé des différences marquées dans la prévalence du paludisme selon les types d'animaux possédés, tant dans les modèles bruts que dans les modèles ajustés. La possession de poulets par les ménages était associée à 3-7 (IC : 0-6, 7-1) infections *P. falciparum* supplémentaires pour 100 personnes, tandis que la possession de bovins était associée à 9-6 (-15-8, -3-5) infections en moins pour 100 personnes, même après prise en compte de l'utilisation de moustiquaires, de la richesse et de la structure de l'habitat.

Interprétation : Dans cette première enquête nationale sur la possession d'animaux par les ménages et la prévalence de *P. falciparum* en RDC, l'association protectrice conférée par la possession de bétail suggère que les interventions de zooprophylaxie pourraient avoir un rôle à jouer en RDC, peut-être en détournant l'alimentation d'*An. gambiae* de l'homme. Les études sur les pratiques d'élevage et les comportements associés des moustiques peuvent révéler des opportunités pour de nouvelles interventions contre le paludisme.

Financement : Cette étude a bénéficié d'un soutien partiel des NIH (F30AI169752 pour CEM, R01AI139520 pour JBP, R01AI129812 pour KM, AKT, et K24AI134990 pour JJJ). Ce travail a été soutenu en partie par la Fondation Bill & Melinda Gates (OPP1161913 à ME, KM). Les bailleurs de fonds n'ont joué aucun rôle dans la conception de l'étude, la collecte des données, l'analyse et l'interprétation des données, la préparation du manuscrit ou la décision de le soumettre.
