# Supplementary material for: Association between domesticated animal ownership and Plasmodium falciparum parasite prevalence in the Democratic Republic of the Congo: a national cross-sectional study
Source: Lancet Microbe. 2023 Jul;4(7):e516–23. doi: 10.1016/S2666-5247(23)00109-X (PMC10319634; doi:10.1016/S2666-5247(23)00109-X)
Supplement: Lingala translation of the abstract [file mmc2.pdf]

THE LANCET
Microbe

Supplementary appendix 2

This translation in Lingala was submitted by the authors and we reproduce it as supplied. It has not been peer reviewed. *The Lancet*'s editorial processes have only been applied to the original in English, which should serve as reference for this manuscript.

Bolimboli oyo na lingala ekotisamaki na bakomi pe biso to bandeli bokotisi lolenge elekelaki. Bolimboli oyo etalamaki te na ba ninga misusu. Ba makambo oyo ya boponomi ba Lancet etalisamaki te na Mukanda ya yambo na anglais oyo esengelaki kozala lokola ndakisa pona Mukanda ya manuscrit oyo.

Supplement to: Morgan CE, Topazian HM, Brandt K, et al. Association between domesticated animal ownership and *Plasmodium falciparum* parasite prevalence in the Democratic Republic of the Congo: a national cross-sectional study. *Lancet Microbe* 2023; published online May 31. https://doi.org/10.1016/S2666-5247(23)00109-X.

Moto ya likambo : Lisangá kati na bozwi ya bibwele ya ndako mpe bopanzani ya parasite ya malaria *Plasmodium falciparum* na République démocratique du Congo: Enquête nationale transversale

Camille E. Morgan, BSPH[1*], Hillary M. Topazian, PhD[2], Katerina Brandt, BA[3], Cedar Mitchell, PhD[4], Melchior Kashamuka Mwandagalirwa, MSPH[5], Jérémie Muwonga, PhD[6], Eric Sompwe, MD, PhD[7,8], Jonathan J. Juliano, MD[1,9], Thierry Bobanga, MD, PhD[10], Antoinette Tshefu, MD, PhD[5], Michael Emch, PhD[3], Jonathan B. Parr, MD, MPH[9]

Na Bokuse

Tina : Bozwi ya ndako ya banyama ya bilanga ya ndako ezali likambo oyo bayekoli mingi te ya zinga zinga ya bato oyo ezali na bopusi na bizaleli ya koswa ngungi mpe bopanzani ya malaria, mpe ezali eteni ya ntina ya nkita mpe bomoi ya ekolo na bisika oyo malaria ezali mingi. Na kotalaka mingi République Démocratique du Congo (RDC), esika 12% ya ba cas ya malaria elobami na mokili mobimba mpe esika ba vecteurs anthropophiles *Anopheles gambiae* ezali mingi, boyekoli oyo elukaki kososola bokeseni na bopanzani ya *Plasmodium falciparum* engebene na ezalela ya bozwi ya sambo ya bato mingi banyama ya ndako.
Lolenge ya kosalela : Na kosalelaka ba données ya enquête ya batu ya âge 15-59 ans na Enquête Démographique et Santé (DHS) ya RDC oyo euti koleka (2013-14) mpe PCR quantitative en temps réel (qPCR) ya Plasmodium oyo esalemaki liboso, to estimé différences na prévalence ya *P. falciparum* basé na ntina ya bozwi ya ndako ya bangombe, nsoso, mpunda, libata, bantaba, bampate, mpe ngulu. Tosalelaki ba graphiques acycliques dirigés pona ko contrôler pona ba confondants lokola âge, genre, bomengo, ndako ya mikolo oyo, bosaleli ya ba ITN, propriété ya mabele ya bilanga, province, pe esika ya mboka.
Biyano : Na ba participants 17.701 oyo bazalaki na ba résultats ya qPCR mpe ba données covariées, pene na ndambo na bango bazalaki na nyama ya ndako, tomonaki bokeseni ya makasi na prévalence ya malaria na ba types ya ba animaux de compagnie oyo bazalaki na yango, ezala na ba modèles bruts koleka na ba modèles ajustés. Bozwi ya nsoso na ndako ezalaki na boyokani na 3.7 (CI: 0.6, 7.1) ya ba infections ya *P. falciparum* ya kobakisa na bato 100, nzokande bozwi ya bangombe ezalaki na boyokani na 9.6 (-15.8, -3.5) ba infections moke na bato 100, ata sima ya ko contrôler mpo na usage net, bomengo ya ndako mpe structure.
Kolimbola : Na enquête nationale oyo ya liboso ya bozwi ya banyama ya ndako pe bopanzani ya *P. falciparum* na RDC, lisanga ya bobateli oyo epesami na bozwi bibwele epesi likanisi ete ba interventions ya zooprophylaxie ekoki kozala na rôle ya kosala na RDC, peut-être -ezala na ko détourner alimentation électrique ya An. gambiae ya moto. Boyekoli ya misala ya kobokola mpe bizaleli oyo esangisi yango ya ngungi ekoki komonisa mabaku ya misala ya sika ya malaria.
Mbongo ya kosala boyekoli oyo ezwaki lisungi ya ndambo uta na NIH (F30AI169752 mpo na CEM, R01AI139520 mpo na JBP, R01AI129812 mpo na KM, AKT, mpe K24AI134990 mpo na JJJ). Mosala oyo ezwaki lisungi ya ndambo na Fondation Bill & Melinda Gates (OPP1161913 na ME, KM). Ba fournisseurs bazalaki na rôle te na conception ya études, collecte ya ba données, analyse mpe interprétation ya ba données, préparation ya manuscrit, to décision ya kotinda yango.
