# Supplementary material for: Association between domesticated animal ownership and Plasmodium falciparum parasite prevalence in the Democratic Republic of the Congo: a national cross-sectional study
Source: Lancet Microbe. 2023 Jul;4(7):e516–23. doi: 10.1016/S2666-5247(23)00109-X (PMC10319634; doi:10.1016/S2666-5247(23)00109-X)
Supplement: Supplementary appendix 3 [file mmc3.pdf]

THE LANCET
Microbe

Supplementary appendix 3

This appendix formed part of the original submission and has been peer reviewed. We post it as supplied by the authors.

Supplement to: Morgan CE, Topazian HM, Brandt K, et al. Association between domesticated animal ownership and *Plasmodium falciparum* parasite prevalence in the Democratic Republic of the Congo: a national cross-sectional study. *Lancet Microbe* 2023; published online May 31. https://doi.org/10.1016/S2666-5247(23)00109-X.

Supplementary Material

Table of Contents

SUPPLEMENTARY FIGURE 1 2
SUPPLEMENTARY FIGURE 2 6
SUPPLEMENTARY FIGURE 3 7
SUPPLEMENTARY FIGURE 4 8
SUPPLEMENTARY FIGURE 5 9
SUPPLEMENTARY FIGURE 6 11
SUPPLEMENTARY TABLE 1 12

Supplementary Figure 1. Directed acyclic graphs for cattle (A) and chickens (B), underpinning theoretical approach to adjusted model covariate selection for large, grazing animals (cattle and horses) and smaller, peri-household animals (chickens, goats, ducks, sheep, and pigs).

A.

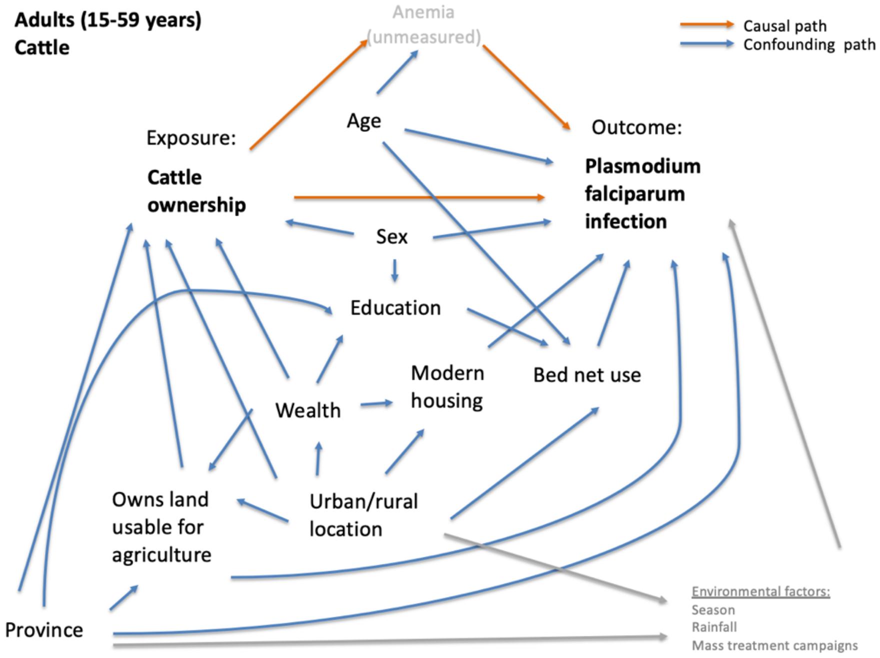

For the theoretical basis of our model selection, we began with a directed acyclic graph to chart the relationships between animal ownership and *P. falciparum* infection.

The following DAGitty code can be used to reproduce the DAG at http://www.dagitty.net/dags.html.

```
dag {
"bed net use" [pos="0.001,-0.687"]
"cattle ownership" [exposure,pos="-1.781,-0.895"]
"land for ag" [pos="-1.375,0.575"]
"modern housing" [pos="-0.261,0.135"]
"pfldh+" [outcome,pos="0.766,-0.901"]
Rurality [pos="0.418,0.581"]
age [pos="-0.528,-1.160"]
anemia [pos="-0.545,-1.633"]
education [pos="-1.070,-0.492"]
province [pos="-2.083,0.432"]
Gender [pos="-0.716,-0.785"]
wealth [pos="-1.084,0.081"]
"bed net use" -> "pfldh+"
"cattle ownership" -> "pfldh+"
"cattle ownership" -> anemia
"land for ag" -> "cattle ownership"
```

```
"land for ag" -> "pfldh+"
"modern housing" -> "bed net use"
"modern housing" -> "pfldh+"
Rurality -> "bed net use"
Rurality -> "land for ag"
Rurality -> "modern housing"
Rurality -> "pfldh+"
age -> "bed net use"
age -> "cattle ownership"
age -> "pfldh+"
anemia -> "pfldh+"
education -> "bed net use"
education -> "modern housing"
province -> "cattle ownership"
province -> "land for ag"
province -> "pfldh+"
province -> education
Gender -> "pfldh+"
Gender -> education
wealth -> "cattle ownership"
wealth -> "modern housing"
wealth -> education
}
```

We determined the following options for sufficient adjustment sets for the cattle analysis:

- Rurality, age, bed net use, land for ag, modern housing, province, gender
- Rurality, age, education, land for ag, modern housing, province, gender
- Age, land for ag, province, wealth

We selected a preferred adjustment set based on the following criteria: 1) covariates more closely related to the outcome rather than the exposure, as this reduces collinearity; and 2) preferring covariates causally associated with malaria (e.g. treated bed net use, modern housing). For this reason, we began with option 1.

Substantive expertise among authors supported inclusion of wealth as well, as higher wealth is associated with cattle ownership and lower malaria prevalence, and is a cited alternate explanation for an association. Adjustment for wealth opened no new backdoor paths, resulting in covariates: rurality, age, bed net use, land for ag, modern housing, province, gender, and wealth.

We then sought to reduce the model by evaluating change in estimate and precision, in order to present prevalence differences, which are preferred to ratio measures for interpretability and conveying public health significance. We used change in estimate as the criteria for this evaluation, as this is a substantive question, not a statistical one, and thus is preferred to likelihood ratio tests or comparing AICs. We conducted this comparison using the log-binomial models (which produce prevalence ratios).

As a result of the comparison, we decided to proceed with a model with the final list of covariates: gender, rurality, wealth, modern housing, and bed net use. As there was not a substantive difference in estimate with the model that included agricultural land ownership, we proceeded with these covariates as it allows for the same set for all animal types (see discussion of Supplementary Figure 1b, below).

B.

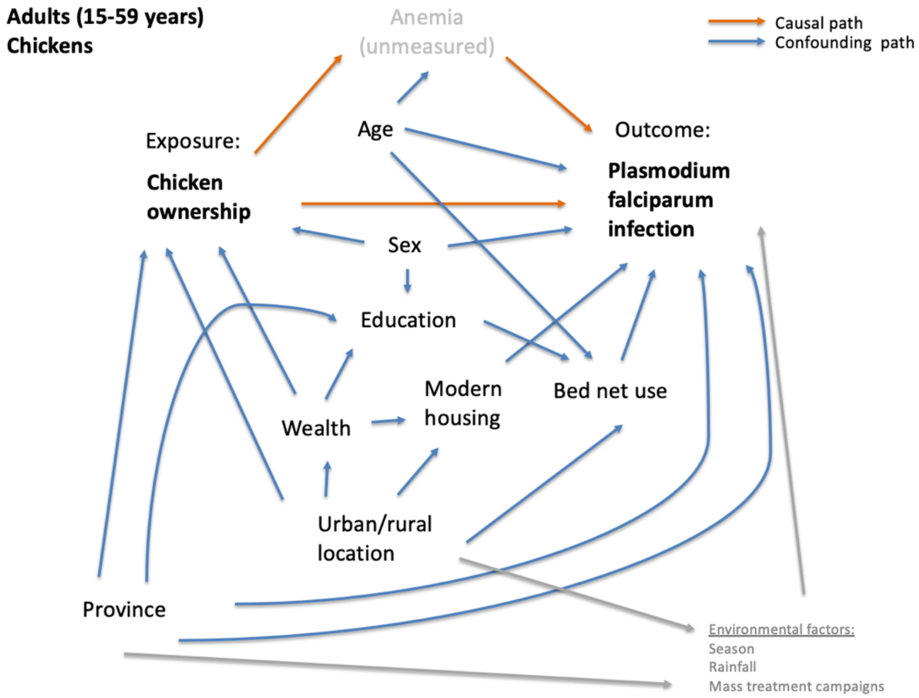

Similarly, for smaller animals,

```
dag {
"Bed net use" [pos="0.250,0.730"]
"Modern housing" [pos="-0.470,1.151"]
"Owns chickens" [exposure,pos="-1.781,-0.293"]
"Pfldh+" [outcome,pos="0.755,-0.364"]
"Urban/rural" [pos="0.534,1.585"]
"household wealth" [pos="-1.392,1.196"]
Anemia [latent,pos="-1.004,-1.419"]
Education [pos="-0.609,0.319"]
Province [pos="-2.199,0.504"]
Gender [pos="-0.260,0.141"]
age [pos="-0.615,-0.921"]
"Bed net use" -> "Pfldh+"
"Modern housing" -> "Bed net use"
"Modern housing" -> "Pfldh+"
```

```
"Owns chickens" -> "Pfldh+"
"Owns chickens" -> Anemia
"Urban/rural" -> "Bed net use"
"Urban/rural" -> "Modern housing"
"Urban/rural" -> "Owns chickens"
"Urban/rural" -> "Pfldh+"
"Urban/rural" -> "household wealth"
"household wealth" -> "Owns chickens"
"household wealth" -> Education
Anemia -> "Pfldh+"
Education -> "Bed net use"
Province -> "Owns chickens"
Province -> "Pfldh+"
Province -> Education
Gender -> "Pfldh+"
Gender -> Anemia
Gender -> Education
age -> "Bed net use"
age -> "Owns chickens"
age -> "Pfldh+"
age -> Anemia
}
```

We identified the following sufficient set of covariates:

- Bed net use, Modern housing, Province, Gender, Urban/rural, age
- Education, Province, Gender, Urban/rural, age
- Province, Urban/rural, age, household wealth

We followed a similar model selection process for the models with smaller animals as with the larger animals. We added wealth, as it is a cited alternate explanation and did not open backdoor paths, and we sought to reduce the model by comparing change in estimates. No substantive change was noted in the prevalence ratios, so we proceeded with the reduced model in order to present prevalence differences.

Supplementary Figure 2. Flowchart of study participants

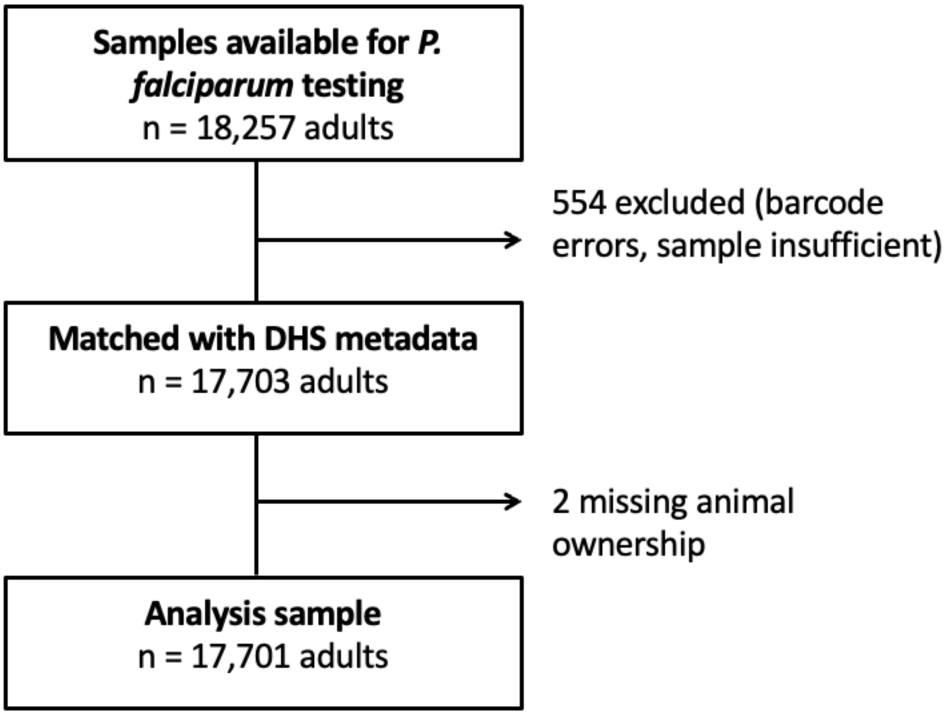

Supplementary Figure 3. Distribution of number of animals owned by the households of participants.

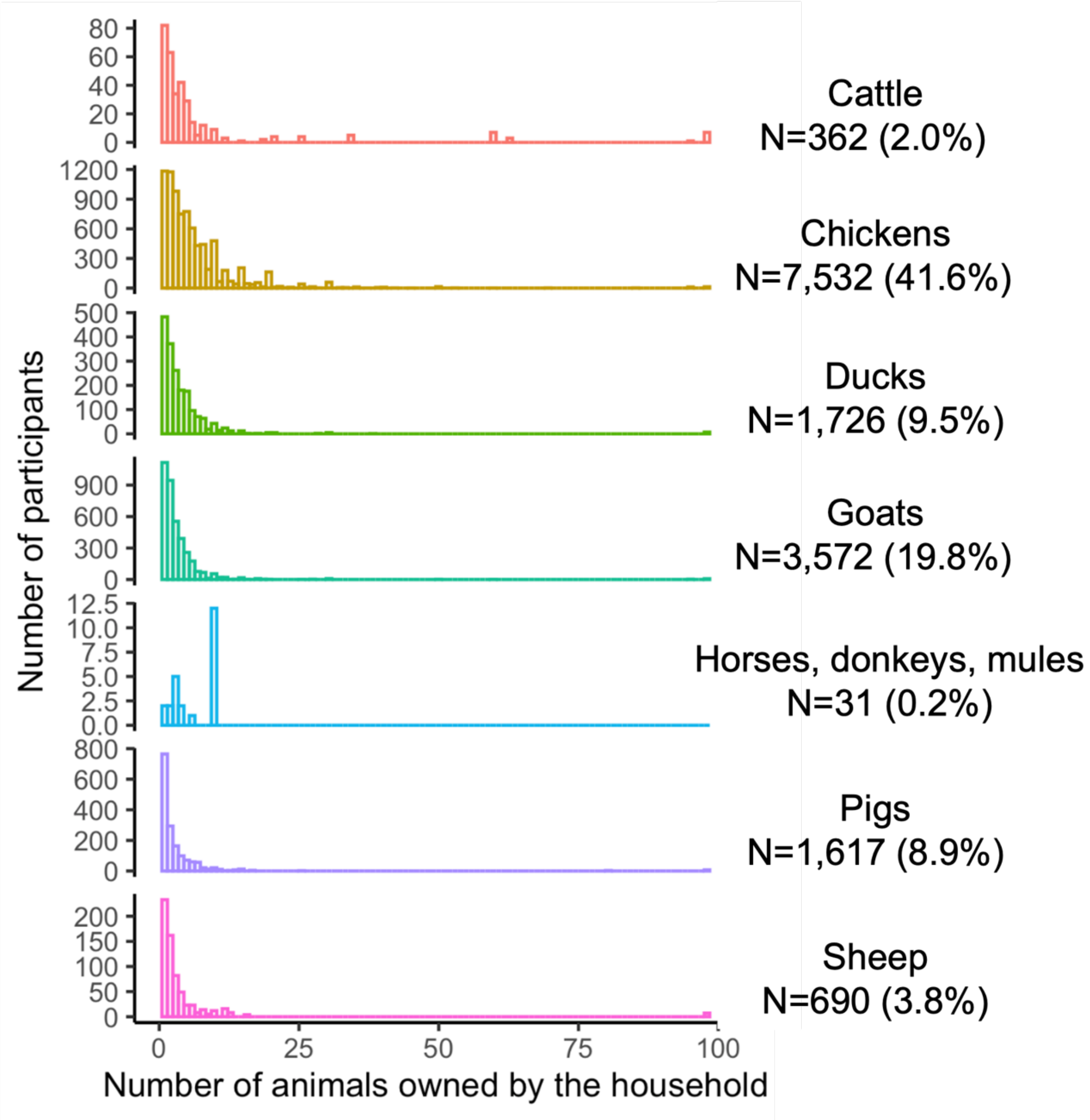

Supplementary Figure 4. Scaled Euler diagram approximating the proportional overlap of different animals owned. Horses, donkeys, or mules are removed due to low counts. Created using R package 'eulerr'.

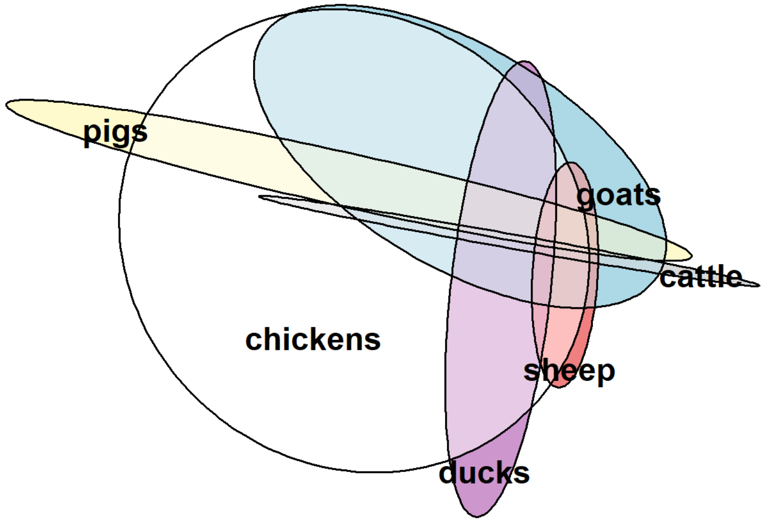

Supplementary Figure 5. Predicted regional distribution of ownership of cattle, chickens, goats, ducks, sheep, and pigs (A-F), and DHS clusters with at least one household with horses or donkeys (G).

A1 (cattle), B1 (chickens), C1 (goats), D1 (ducks), E1 (sheep), and F1 (pigs) represent the predicted percentage of households in a given area that own any of the given livestock. Panels A2, B2, C2, D2, E2, and F2 represent the respective standard errors of the predicted surfaces as the percentage of households who own the livestock type. Ordinary kriging was used to create each of the six predicted surfaces and their respective uncertainty surfaces individually, using the 'gstat' package in R 4.2.1 (Pebesma 2004; Gräler, Pebesma, & Heuvelink 2016). For each animal type, the semivariogram was fit based on the percentage of households who owned at least one animal of the given type at each Enumeration Area in the DHS dataset. Then, the fitted model was used to predict ownership of the animal type across the DRC. These surfaces are an easily interpretable visualization of household animal ownership across the country in 2013-2014. Cattle ownership is mostly clustered in the southwest, on the eastern border, and in the southeastern tip bordering Zambia (A1). Chickens are owned throughout the DRC (B1). Goats are the most commonly owned animal after chickens, and higher rates of ownership are observed in the southern and northeastern parts of the country (C1). Duck ownership is mainly distributed in the northern half of the country (D1). Sheep ownership appears to be low and sporadically distributed throughout the country (E1). Pig ownership is distributed throughout the country (F1). We chose not to create ownership prediction surfaces for horses/donkeys because of low numbers of households owning these animals (N=31 out of 18,091 households, weighted) but instead highlight the DHS clusters with at least one household that reported them (G).

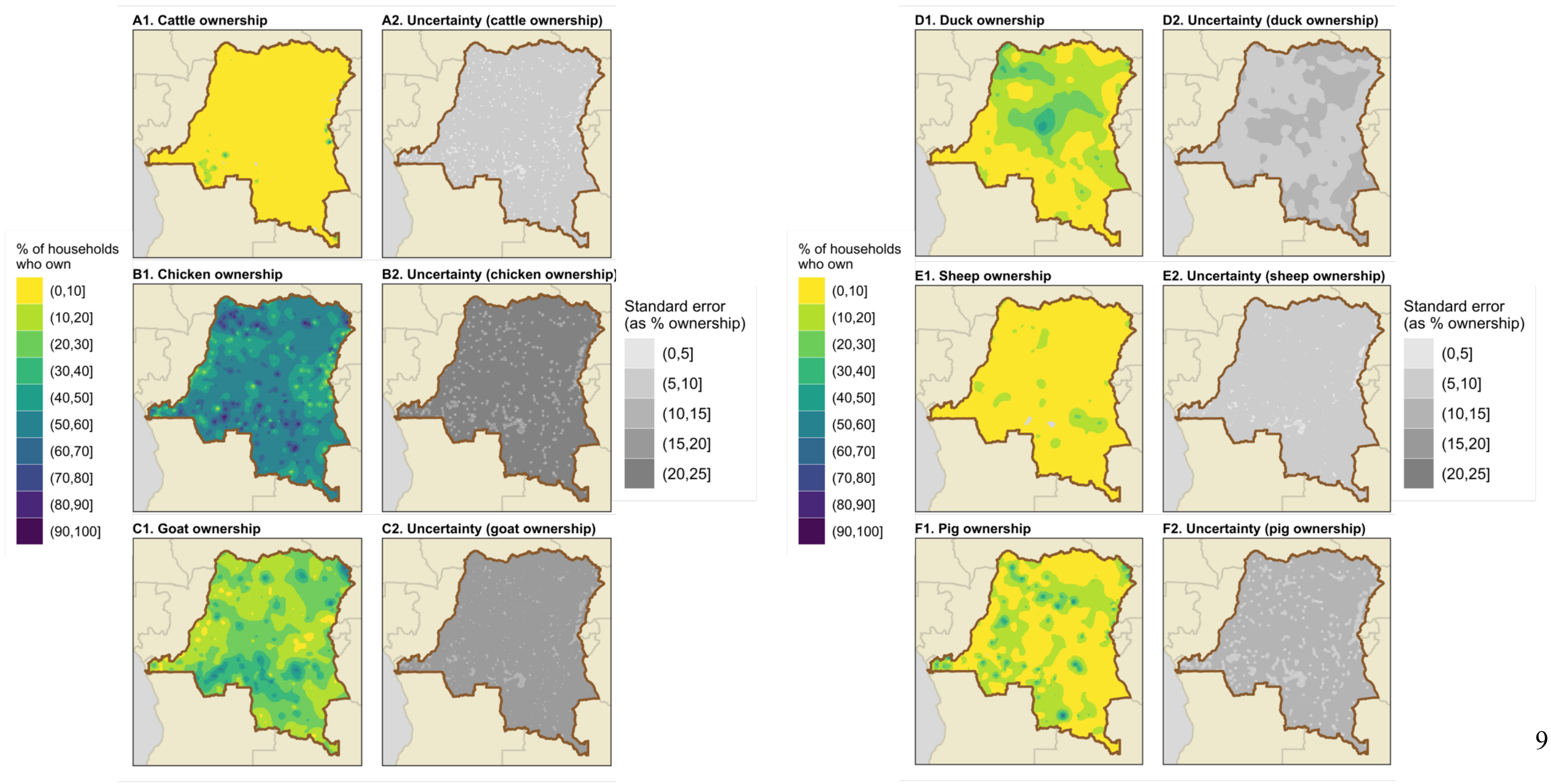

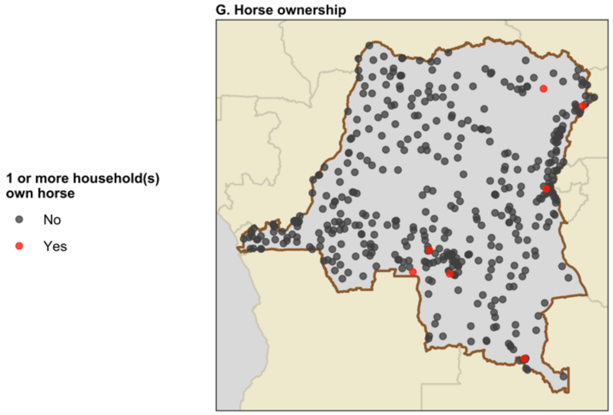

References

Pebesma, E.J., 2004. Multivariable geostatistics in S: the gstat package. Computers & Geosciences, 30: 683-691.
Benedikt Gräler, Edzer Pebesma and Gerard Heuvelink, 2016. Spatio-Temporal Interpolation using gstat. The R Journal 8(1), 204-218

Supplementary Figure 6. *P. falciparum* prevalence by overlapping animal ownership among those who owned at least two animal types. Prevalence estimates are weighted to account for the survey sampling design.

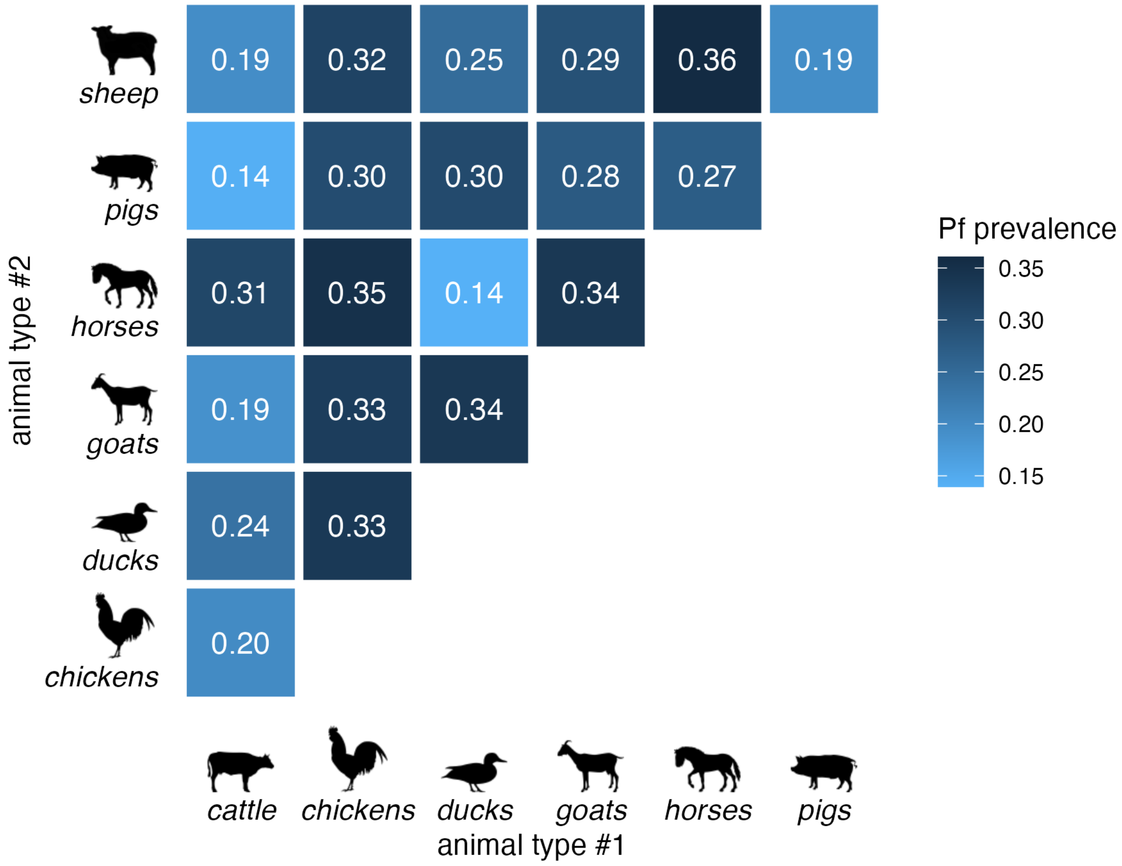

Supplementary Table 1. Effect Measure Modification

Identity link regression models to calculate prevalence differences did not converge when the modifier term was added, so logistic regression was used to address convergence. Generalized estimating equation models (GEE) are weighted via a survey design. Agricultural land ownership was a self-reported binary response on the original survey.

**Modifier**	**Animal exposure**	***Pf*, n***	**No *Pf*, n***	**Adjusted odds ratio†**
Agricultural land ownership	Cattle	48	264	0.39 (0.23, 0.65)
	No cattle	3697	6905	Ref
No agricultural land ownership	Cattle	14	35	1.60 (0.95, 2.70)
	No cattle	1865	5261	Ref
Agricultural land ownership	Chickens	2049	3687	1.17 (0.99, 1.39)
	No chickens	1695	3480	Ref
No agricultural land ownership	Chickens	547	1248	1.16 (0.88, 1.53)
	No chickens	1332	4049	Ref

*Weighted counts, *Pf* = *P. falciparum* infection; No *Pf* = No *P. falciparum* infection
†Adjustment set includes gender, bed net, modern housing, wealth, rurality.
